# Developmental Changes of Prefrontal Activation in Humans: A Near-Infrared Spectroscopy Study of Preschool Children and Adults

**DOI:** 10.1371/journal.pone.0025944

**Published:** 2011-10-12

**Authors:** Yuki Kawakubo, Toshiaki Kono, Ryu Takizawa, Hitoshi Kuwabara, Ayaka Ishii-Takahashi, Kiyoto Kasai

**Affiliations:** 1 Department of Child Neuropsychiatry, Graduate School of Medicine, University of Tokyo, Tokyo, Japan; 2 Department of Neuropsychiatry, Graduate School of Medicine, University of Tokyo, Tokyo, Japan; Chiba University Center for Forensic Mental Health, Japan

## Abstract

Previous morphological studies indicated that development of the human prefrontal cortex (PFC) appears to continue into late adolescence. Although functional brain imaging studies have sought to determine the time course of functional development of the PFC, it is unclear whether the developmental change occurs after adolescence to adulthood and when it achieves a peak because of the narrow or discontinuous range in the participant's age. Moreover, previous functional studies have not focused on the anterior frontal region, that is, the frontopolar regions (BA9/10). Thus, the present study investigated the developmental change in frontopolar PFC activation associated with letter fluency task by using near-infrared spectroscopy (NIRS), in subjects from preschool children to adults. We analyzed the relative concentration of hemoglobin (ΔHb) in the prefrontal cortex measured during the activation task in 48 typically-developing children and adolescents and 22 healthy adults. Consistent with prior morphological studies, we found developmental change with age in the children/adolescents. Moreover, the average Δoxy-Hb in adult males was significantly larger than that in child/adolescent males, but was not true for females. These data suggested that functional development of the PFC continues into late adolescence. Although the developmental change of the frontopolar PFC was independent of gender from childhood to adolescence, in adulthood a gender difference was shown.

## Introduction

The pattern of development and maturation of the human prefrontal cortex (PFC) from childhood through early adulthood is an important research question in neuroscience. The activity of the catechol-o-methyltransferase (COMT) enzyme that modulates dopamine levels in the PFC increases from the neonate through to adulthood [1], consistent with the critical role of dopamine in modulating normal PFC function [2]. Previous morphological studies have used postmortem brains and MRI to indicate that development appears to continue into late adolescence in terms of synaptic density [3], gray matter volume [4], [5] and cortical thickness [6].

Functional brain imaging studies have also sought to determine the time course of functional development of the PFC, although the findings have been equivocal. A positron emission tomography (PET) study showed that glucose metabolism at 4 years and 9–10 years was at a high plateau and after 9–10 years began to decline and gradually reaches adult values by 16–18 years [7]. Some functional MRI (fMRI) studies showed that the activation of the dorsolateral PFC (DLPFC) increased with age during the declarative memory task for 8–24 year-olds [8], and that the greater activation in adults than in adolescents during the Stroop task for 7–22 year-olds [9]. Others indicated that DLPFC was more active in children (9–12 year-olds) than in adults (20–30 year-olds) in the go/no-go task [10], and that adolescents (14–17 year-olds) showed greater activation than children (8–13 year-olds) and adults (18–30 year-olds) in the saccade task [11]. The ventrolateral PFC (VLPFC) was activated in adults only during the go/no-go task, but not in children (8–12 year-olds) [12], while children (8–13 year-olds) demonstrated greater activation than adults (19–48 year-olds) in the verbal fluency task [13]. In a near-infrared spectroscopy (NIRS) study, both adults and preschool children (5–6 year-olds) increased oxyhemoglobin (oxyHb) in the lateral PFC (LPFC) during the working memory task and the activation of LPFC was larger and broader in children than in adults, although children were not directly compared with adults [14]. Another study using the Stroop task, however, showed that the oxyHb responses in the young adults were greater and faster than those in children (7–13 year-olds), and reported that the DLPFC activation increased with age [15]. To summarize the above findings, previous studies have been mixed regarding in which life stage (childhood, adolescence, adulthood) the LPFC activation becomes largest.

The disagreement in functional brain imaging studies might be attributed to participant's age, task demands or the experimental paradigm, such as event-related design or blocked design. Since few studies included participants with a broad range in age from childhood to adulthood, it is unclear whether the developmental change occurs between adolescence and adulthood and when it achieves its peak. Moreover, although previous studies have investigated the anterior frontal region, that is, the frontopolar regions (BA9/10), they have not focused on it. The frontopolar regions have a higher-order integrative prefrontal function [16] and comparative studies of humans and apes [17] suggested that they have enlarged and become specialized during hominid evolution. The frontopolar regions might coordinate VLPFC and DLPFC functions in order to achieve task goals or maximize task performance [18]–[20], and might evaluate internally generated information [21]. Because the frontopolar cortex is located in the vicinity of air-filled spaces of the nasal cavity, the corresponding magnetic susceptibility differences at air–tissue or bone–tissue interfaces result in severe distortions and regional signal losses in long-TE gradient-echo images, particularly for ultrafast imaging techniques such as echo-planar imaging in a high magnetic field. Therefore, such observation without signal losses in the frontopolar PFC might be one of the reasons for the superiority of NIRS.

NIRS is one of the most promising noninvasive functional neuroimaging tools to allow comparative evaluation of cortical hemodynamic response for children and individuals with psychiatric disorders. NIRS can measure the signals reflecting relative concentrations change of oxy-hemoglobin (Δoxy-Hb) and deoxy-hemoglobin (Δdeoxy-Hb), which are assumed to reflect regional cerebral blood volume (rCBV). While fMRI and PET have an excellent spatial resolution, they are limited in that they require a large apparatuse that prevents their use in bedside settings for diagnostic and treatment purposes. In contrast, NIRS is a neuroimaging modality that, for the following reasons is especially suitable for assessing the PFC of infants [22], children [14], [15] and psychiatric disorders [23]–[28] because NIRS is relatively insensitive to motion artifacts, it can be applied to experiments that might cause some motion of the subjects, such as vocalization. Second, the subject can be examined in a natural sitting position, without any surrounding distraction. Third, the cost is much lower than other neuroimaging modalities and the set-up is very easy. Fourth, as the test-retest reliability at weekly and monthly intervals has demonstrated [29], [30], NIRS can be applied to longitudinal assessment following intervention. Fifth, the high temporal resolution of NIRS is useful in characterizing the time course of prefrontal activity [23]–[25].

By simultaneous measurements with other methodologies, it has been shown that the Δoxy-Hb measured by NIRS correlates with the rCBF change in 15H2O PET [31] and the blood oxygenation level-dependent [32] signal in fMRI [33]. In other fMRI studies [32], [34], [35], in which the Δoxy-Hb was not analyzed, the Δdeoxy-Hb in NIRS has been correlated with the BOLD signal.

Moreover, previous studies showed that the verbal fluency test is a valid cognitive activation task to evaluate ΔHb in PFC using NIRS [24]–[26], [28], [31]. In NIRS studies recording the ΔHb during several tasks for the same subject group, the smaller-than-normal Δoxy-Hb during the cognitive tasks involving primarily the PFC, such as the letter fluency test and the random number generation task, was task specific in schizophrenia, i.e., this was not evident during other tasks, such as the sequential finger-to-thumb task [36], or the finger tapping task [24]. These findings suggested that the Δoxy-Hb reflected the neural activation but not general or nonspecific factors, such as impaired vascular responsiveness irrespective of neural activation or optical pathlength.

Thus, the present study investigated the developmental change in frontopolar PFC activation associated with the letter fluency task by using NIRS, in a group of subjects that included preschool children to adults.

## Methods

### 1) Subjects

Subjects were 48 typically-developing children and adolescents (22 male and 26 female; age range, 5–18 years; mean age, 10.9; mean IQ, 106.2) and 22 healthy adults (11 male and 11 female; age range, 21–37 years; mean age, 27.3; mean IQ, 113.1) (Table 1). Participants were mainly recruited from college students, hospital staff, their acquaintances and children, and those who volunteered for participating through the laboratory's web site. When siblings or twin pairs participated in this study, only one was randomly selected and included in the data analysis (five children were from siblings and 23 children from twin pairs). As shown in the results section, the twin subjects and non-twin subjects did not significantly differ in ΔHb. The exclusion criteria were neurological illness, traumatic brain injury with any known cognitive consequences or loss of consciousness for more than 5 minutes, a history of electroconvulsive therapy, and alcohol/substance abuse or addiction. An additional exclusion criterion was a history of psychiatric disease or a family history of axis I disorder in their first-degree relatives. IQs were evaluated with the WISC-III or WAIS-R. All participants were right-handed as based on the Edinburgh Inventory [37] and were native Japanese speakers.

**Table 1:** Mean of age and IQ in each group.

	Child/adolescent	Adult		
	Male	Female	Male	Female
n	22	26	11	11
age	9.9±2.7	11.7±3.8	26.5±5.7	28.2±5.5
(range)	(5.8∼17.1)	(5.5∼18.6)	(21.4∼37.4)	(21.8∼36.4)
IQ^a^	108.5±13.5	104.2±11.0	115.9±11.4	110.4±10.1
(range)	(81∼137)	(82∼123)	(94∼128)	(92∼125)

a For participants aged 15 and under IQ was evaluated with the WISC-III, for participants aged 16 and over it was estimated by four subtests of the WAIS-R.

### 2) Ethics

The ethical committee of the Faculty of Medicine, University of Tokyo approved this study (No. 630-5). All adult participants gave written informed consent. All child participants gave informed assent and their parents gave written informed consent.

### 3) Activation task

The activation task consisted of a 30 sec rest, a 30 sec letter fluency task and a 30 sec rest. In the letter fluency task, participants were asked to say as many words that began with a Japanese character /a/ as they could. The participants sat on a chair with their eyes open and held their hands on their lap throughout the measurement. The auditory cues were presented at the start and end of the letter fluency task or rest. Hemoglobin concentration changes were measured during the activation task. The activation task was similar to that in previous studies [24], [26], but 3 changes were introduced to make the task suitable for children: 1) In the pre- and post-task participants were silent instead of repeating moras; 2) The time period of the letter fluency task and post-task was shortened to 30 sec from 60 sec; 3) Only a single mora was used in the letter fluency task. The number of words generated during the letter fluency task was determined as a measure of task performance.

### 4) NIRS measurement

**Figure 1:**
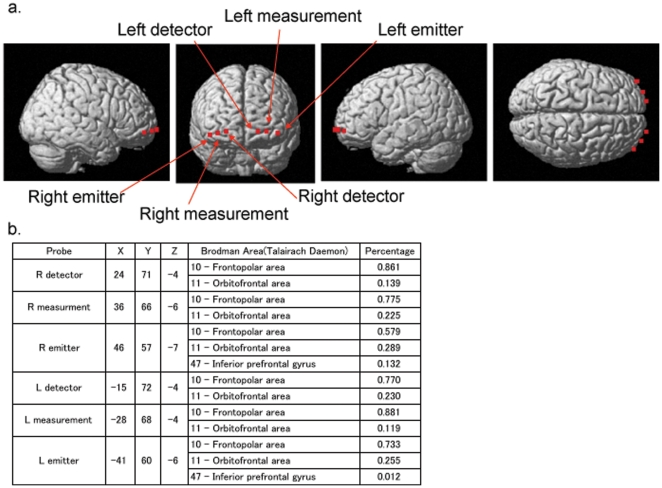
The probe positions and the measurement areas. a:The correspondence of the probe positions and the measurement areas on the cerebral cortex. b: The locations of NIRO probe were probabilistically estimated and anatomically labeled in the standard brain space (Brodmann's Area) according to [39].

Δoxy-Hb and Δdeoxy-Hb was measured using a 2-channel NIRS machine (NIRO200, Hamamatsu Photonics, Inc) at three wavelengths of near-infrared light (775, 810, 850 nm). The measurement principles were based on the modified Beer-Lambert law, which calculates Δoxy-Hb and Δdeoxy-Hb from the light attenuation change at a given measured point. Δoxy-Hb and Δdeoxy-Hb values include a differential pathlength factor and are given in units of mMmm. Each of the two probes consisted of an emitter and a detector separated by 4 cm. The two NIRS probes were placed on the subject's prefrontal regions and secured using double-sided adhesive tape such that the detectors were positioned at Fp1 and Fp2 with the emitters positioned 4 cm on the lateral side of the detectors along the T3–T4 line, according to the international 10/20 system. The machine measured ΔHb approximately 2–3 cm beneath the scalp, i.e., the cortical surface area [31], [34]. NIRS probes measured oxygenation at the Brodmann's area 10 (figure 1). The correspondence of the probe positions and the measurement areas on the cerebral cortex was confirmed by superimposing the measurement positions on a magnetic resonance image of a three-dimensionally reconstructed cerebral cortex for a healthy adult. The locations of NIRO probe were probabilistically estimated and anatomically labeled in the standard brain space (Brodmann's Area) according to [38]. Also, the correspondence was supported by a multisubject study of anatomical cranio-cerebral correction via the international 10–20 system [39]. The sampling time for the recording was 0.5 sec. Baseline correction was made by using the average ΔHb value during the first 30 sec rest, and then the average ΔHb value during the 30 sec task period was calculated in each hemisphere.

### 5) Statistical Analysis

A 2-way ANOVA with age (child/adolescent, adult) and gender (male, female) as the between-subjects factors used to analyze task performance.

For the mean ΔHb during the 30 sec task period, a 3-way ANCOVA was performed with age (child/adolescent, adult) and gender (male, female) as the between-subjects factors, hemisphere (left, right) as the within-subjects factor and task performance as a covariate. When the sphericity assumption was violated, Greenhouse-Geisser correction was applied and the associated epsilon was reported. For post-hoc analysis, the mean ΔHb of the hemispheres was used as the dependent variable with task performance as a covariate and statistically significant level was defined as p<.025 (Bonferroni correction).

We calculated the Pearson's correlation between the average ΔHb and task performance and age separately for each gender in the child/adolescent and adult groups. Second, the comparison of correlation coefficients between male and female was performed.

## Results

### 1) Task performance

The mean number of words generated during the letter fluency task was: 4.32 (SD = 2.61) for male; 4.38 (SD = 2.23) for female in the child/adolescent group, and 9.27 (SD = 2.90) for male; 8.55 (SD = 1.51) for female in the adult group. A main effect of age was significant (F(1,66) = 55.20, p<.001), but the effect of gender and the interaction were not significant (gender: F(1,66) = .29, p = .59; interaction gender and age: F(1,66) = .42, p = .52).

### 2) Group comparisons of the ΔHb

Twenty-three of the participants in the child/adolescent group were one of a pair of twins. T-test showed that the mean ΔHb was not significantly different between non-twin and twin subject in the child/adolescent group (oxy-Hb: t(46) = .48, p = .63; deoxy-Hb: t(46) = −.90, p = .37), indicating that including one of twins may not have significantly influenced the conclusions of the study.

**Figure 2:**
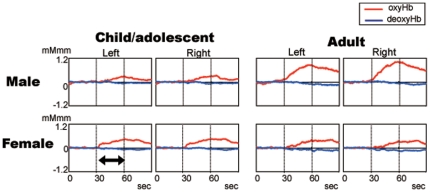
Grand average waveforms of ΔHb during the letter fluency task. Upper: male, lower: female, right: adult group, left: child/adolescent group. Line: red, oxyhemoglobin; blue, deoxyhemoglobin. The period of the activation task is between the two dotted lines.

Figure 2 shows grand average waveforms of hemoglobin concentration changes for each group. The average Δoxy-Hb in the right hemisphere was: mean 0.15 (SD = 0.36) for male; 0.30 (SD = 0.30) for female in the child/adolescent group and 0.65 (SD = 0.45) for male; 0.13 (SD = 0.20) for female in the adult group, and that in the left hemisphere was: mean 0.12 (SD = 0.44) for male; 0.26 (SD = 0.29) for female in the child/adolescent group and 0.57 (SD = 0.49) for male; 0.17 (SD = 0.11) for female in the adult group. For the Δoxy-Hb, there was a significant interaction between age and gender (F(1,65) = 12.27, p<.001). All main effects and other interactions were not significant (age: F(1, 65) = 3.69, p = .059; gender: F(1,65) = 3.54, p = .07; hemisphere: F(1,65) = 1.13, p = .29; interactions of age and hemisphere: F(1,65) = 1.64, p = .21, gender and hemisphere: F(1,65) = 0.97, p = .33, age, gender and hemisphere: F(1,65) = 1.00, p = .32).

**Figure 3:**
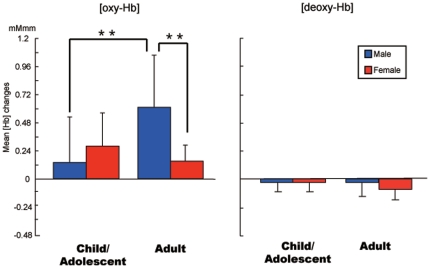
The mean ΔHb of the hemispheres in each group. Left: Δoxy-Hb, right: Δdeoxy-Hb, blue: male, red: female. The average Δoxy-Hb in the adult group was significantly larger than that in the child/adolescent group for male (F(1,30) = 11.55, p<.01). Moreover, for the adult group the average Δoxy-Hb in male was significantly larger than that in female (F(1,19) = 16.15, p<.01).

Since we found a significant interaction between age and gender, we next conducted post-hoc analyses in two ways using the mean Δoxy-Hb of the hemispheres as the dependent variable with task performance as a covariate (Figure 3). First, we compared Δoxy-Hb two age groups separately for each gender. For male, the average Δoxy-Hb in the adult group was significantly larger than that in the child/adolescent group (F(1,30) = 11.55, p<.01). For female, however, it did not reach at a significant level (F(1,34) = 4.69, p = .04). Second, we compared Δoxy-Hb between two gender groups separately for each age group. For the child/adolescent group, there was not a significant difference (F(1,45) = 2.01, p = .16), but in the adult group the average Δoxy-Hb in male was significantly larger than that in female (F(1,19) = 16.15, p<.01).

The average Δdeoxy-Hb in the right hemisphere was mean −.02 (SD = .08) in the child/adolescent group, −.05 (SD = .13) in the adult group, and in the left hemisphere was mean −.03 (SD = .10) in the child/adolescent group, and −.05 (SD = .11) in the adult group. For the Δdeoxy-Hb, there was a significant main effect of hemisphere (F(1,65) = .4.40, p = .04). There were no other significant main effects and any interactions (age: F(1,65) = .57, p = .46; gender: F(1,65) = 1.48, p = .23; interactions of age and gender (F(1,65) = 1.54, p = .22, age and hemisphere: F(1,65) = 2.64, p = .11, gender and hemisphere: F(1,65) = .17, p = .69, age, gender and hemisphere: F(1,65) = 2.69, p = .11).

### 3) Correlation analysis

**Figure 4:**
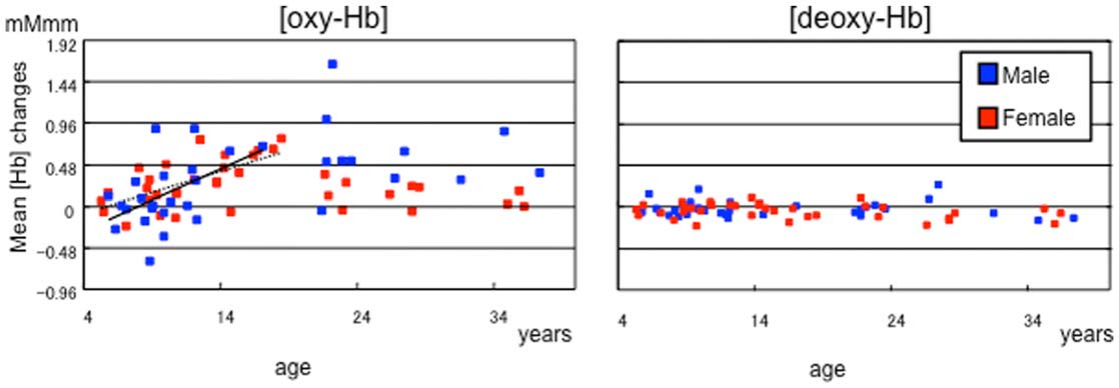
The scatter plots of age and the mean ΔHb of the hemispheres. Left: Δoxy-Hb, right: Δdeoxy-Hb, blue: male, red: female. Contrary to the strongly positive correlation between Δoxy-Hb and age in the child/adolescent group (male: r = 0.50, p = .017; female: r = .67, p<.001), the correlation coefficient was slightly negative but not statistically significant in the adult group.

Since hemoglobin concentrations of both hemispheres did not behave differently as indicated by a lack of significant interactions with hemisphere in the main ANCOVA, we used the mean ΔHb of the hemispheres for the correlational analyses. The the child/adolescent group showed a strongly positive correlation between Δoxy-Hb and age (male: r = 0.50, p = .017; female: r = .67, p<.001), whereas the adult group showed a weak negative correlation which did not reach a significant level (male: r = −.15, p = .65; female: r = −.37, p = .27) (Figure 4). The difference in correlation coefficients between male and female was not significant in the child/adolescent or adult groups (Fisher's r to z transformation; child/adolescent, z = −.76, p = .45; adult, z = .47, p = .64). There were no correlations between the Δoxy-Hb and task performance in the child/adolescent (male: r = −.07, p = .75; female: r = .30, p = .14) or adult groups (male: r = −.59, p = .06; female: r = .05, p = .90).

The Δdeoxy-Hb were not correlated with age in the child/adolescent group (male: r = −.11, p = .62; female: r = −.04, p = .85), or in the adult group (male: r = −.28, p = .41; female: r = −.41, p = .21). The difference in correlation coefficients between male and female was not significant in the child/adolescent or adult groups (child/adolescent, z = .23, p = .82; adult, z = .31, p = .76). There were no correlations between the Δdeoxy-Hb and task performance in the child/adolescent (male: r = −.02, p = .94; female: r = .01, p = .95) or adult groups (male: r = −.19, p = .57; female: r = .31, p = .35).

## Discussion

To our knowledge, this is the first report of developmental changes in frontopolar PFC hemodynamic data from preschool children to adults. First, in the children/adolescent group the Δoxy-Hb during the verbal fluency task was significantly increased with age. Contrary to the strongly positive correlation between prefrontal activation and age in the child/adolescent group, the correlation coefficient was slightly negative but not statistically significant in the adult group. Second, the effect of gender on Δoxy-Hb differed depending on age, where in the adult group the males showed a larger Δoxy-Hb than the females, but in the child/adolescent group there was no difference between the males and the females.

### 1) Developmental change of the frontopolar PFC

Meta-analysis of fMRI [40] and previous multi-channel NIRS studies [24]–[26], [28] showed that frontopolar areas were not the sites of typical activation during letter fluency task, but that widespread regions of the prefrontal cortical surface area and superior temporal regions were recruited. However, comparative studies of humans and apes showed that the frontopolar regions have enlarged and become specialized during hominid evolution [17]. Previous NIRS studies, furthermore, found that the activation of frontpolar region during the letter fluency test were associated with the social functioning in schizophrenia [28]. Thus, even if the frontpolar region was not mainly recruited during the letter fluency test, the activation of this area has important roles of human life because the frontopolar regions have a higher-order integrative prefrontal function [16]. In this study, frontpolar activation increased with age and boys showed smaller activation than men. Although an fMRI study using the verbal fluency task found that activation of the ventrolateral prefrontal cortex (BA44/45) is larger in children than in adults [13], it is not necessarily contradictory that the time course does not agree with previous developmental studies on the function at other regions of the prefrontal cortex. Rather, the present NIRS data was consistent in showing that the BA10 is developed latest in the ontogenetic change and might suggest that the cortical area recruited by the verbal fluency task might shift from the dorso-ventrolateral to the anterior polar region with age. However, this interpretation should be validated in future studies using an instrument with a wider coverage of prefrontal and temporal area.

The results of the correlation analysis in the child/adolescent group suggest that recruitment of the frontopolar PFC during letter fluency tasks increases with age in childhood and adolescence, and that development appears to continue into late adolescence. These results are in agreement with a previous morphological study on the frontopolar PFC [41].

Contrary to the strongly positive correlation between prefrontal activation and age in the child/adolescent group, the correlation coefficient was slightly negative but not statistically significant in the adult group. This was consistent with previous NIRS studies using the letter fluency task, in which the Δoxy-Hb in middle age was smaller than that in young adults [42], [43]. A failure in reaching statistically significant level in this study may be due to the narrow range of the participant's age and the small sample size in the adult group.

### 2) Gender effect on frontopolar PFC activation

In the adult group, the mean Δoxy-Hb during the letter fluency test was larger in the males than in the females. This finding of gender effect on Δoxy-Hb was in agreement with a previous NIRS study using the same task [25]. Mean IQ and mean age were not likely to be main confounding factors, since they were not different between genders.

The gender effect on Δoxy-Hb differed depending on age, where in the child/adolescent group there were no significant differences in correlation coefficients or mean Δoxy-Hb between genders. This developmental course was compatible with other morphological data that reported no gender difference in the fronopolar thickness in subjects 8–20 years of age [41]. In comparison of Δoxy-Hb between the two age groups separately for each gender, the males showed a larger Δoxy-Hb in the adult goup than in the child/adolescent group, but the females showed no difference between the two age groups. Taken together, these results suggested that the developmental change in the frontopolar PFC hemodynamic response until late adolescence occurred independent of gender and that the peak of the Δoxy-Hb was younger and smaller in females than in males. Those findings could be related to a high plateau peak of frontal gray matter at younger and smaller in females than in males [4].

### 3) Methodological issues

First, we used the resting state as the baseline to facilitate applicability of the task for child participants, although we assumed a simple vocalization task for the baseline would be more ideal to derive a pure activation related to the letter fluency task. Therefore, the age-dependent increase in frontopolar PFC activation may reflect an age-dependent increase in brain activity due to vocalization per se or age-dependent hypoperfusion during the baseline period (resting state). PET studies have reported age-dependent decreased oxygen metabolism and regional cerebral blood flow (rCBF) during the resting state in the frontal areas [7], [44]. Thus, it may be possible that the adolescents were hypoperfused during the baseline, and then activation during the task would have been larger compared with that for the younger children. However, this interpretation is incomplete because the age at peak Δoxy-Hb in this study was incongruent with a peak of the rCBF [44] and the glucose metabolic rate [7] in PET studies. Furthermore, it is impossible to distinguish whether these results were due to ‘cognitive development’ or just to ‘phonation development’ and ‘structural development’. Thus, future studies should add a simple vocalization as the referential condition and investigate the structural development

Second, the design used in this study suffered from difference in optical properties of scalp and cortical tissues with age and gender. Adults are expected to have thicker skulls than children, and males' skulls are thicker than females'. Simulation studies on tissue optical properties [45] indicated that the thicker skull contributes toward decreasing amplitude of oxyHb signal. However, the current study showed that that the Δoxy-Hb was largest in the adult male. Thus, although individual difference in optical properties of scalp and cortical tissues is very important in theory, it may not have a substantial effect on the statistical conclusion reported here.

Third, as we measured activation of only the frontopolar regions of the PFC during a letter fluency test in this study, results could not be compared with the activation of other regions and tasks. Thus, a functional control task such as checkerboard rotation or finger tapping tasks and measurement of other regions as reference are needed to compensate individual difference in the tissue optical properties and provide more convincing results in future studies using a multi-channel NIRS machine.

Fourth, we used the cross-sectional design, not the longitudinal one. However, IQ was controlled between the child/adolescent and adult groups for each gender (male: t(31) = −1.56, p = .13; female: t(35) = −1.58, p = .12). Future research with a longitudinal design is necessary for a more comprehensive understanding of developmental change in the PFC.

Fifth, a recent NIRS study showed the influence of skin blood flow on NIRS signals measured on the forehead during a verbal fluency task [46]. This study criticized that frontopolar activation may not represent cortical change but non-cortical physiological signal, which is autonomic control. Thus, it remains possible that our data may at least partially represent the development of autonomic control. Future studies are needed to disentangle contribution of cerebral and skin blood flow on the NIRS signals in various NIRS apparatuses by using, for example, simultaneous measurement of NIRS and fMRI during cognitive activation.

### 4) Conclusion

The present study, which investigated frontopolar PFC activation during the verbal fluency test, suggested that functional development of the area continues to late adolescence. Although the developmental change of the frontopolar PFC was independent of gender from childhood to adolescence, in adulthood a gender difference was shown.
